# A qualitative study on the feasibility and acceptability of a primary care intervention for fear of cancer recurrence

**DOI:** 10.1177/13591053251345579

**Published:** 2025-07-10

**Authors:** Yvonne L Luigjes-Huizer, Melanie PJ Schellekens, Niek J de Wit, Charles W Helsper, Sophie I van Dongen, Anne S van Roozendaal, Rosalie AM van Woezik, Marije L van der Lee

**Affiliations:** 1 Helen Dowling Institute, The Netherlands; 2 Utrecht University, The Netherlands; 3 Tilburg University, The Netherlands

**Keywords:** acceptability, fear of cancer recurrence, feasibility, primary care, qualitative research

## Abstract

Specialized care for fear of cancer recurrence (FCR) exists, but there remains a need for easily accessible interventions for moderate FCR. Recently, a short primary care intervention was shown to be effective in reducing FCR. This study aimed to evaluate the feasibility and acceptability of this intervention by interviewing patients (*n* = 9) and mental health workers (MHWs; *n* = 13). The interviews were analyzed using thematic analysis. The intervention was appreciated for being recognizable, adaptable and stimulating self-management. The combination of an online program and sessions with a mental health worker was also appreciated. The program appeared less suitable for patients lacking certain digital or language skills. The primary care setting was considered very suitable for the intervention, as it addresses both physical and psychosocial complaints. In conclusion, we recommend implementing the program in practice as it is considered feasible and acceptable and has previously been shown effective in reducing FCR.

## Introduction

Globally, cancer incidence is increasing (Sung, 2021), while cancer mortality is decreasing (Santucci et al., 2020), leading to an increasing number of cancer survivors. More than half of cancer survivors experience fear of cancer recurrence (FCR; Luigjes-Huizer et al., 2022b), which has been defined as “fear, worry, or concern relating to the possibility that cancer will come back or progress” (Lebel et al., 2016). FCR can lead to decreased quality of life and increased healthcare costs because of additional consultations with medical professionals (Crist and Grunfeld, 2013; Williams et al., 2021). FCR causes preoccupation, worry, hypervigilance to bodily symptoms (Mutsaers et al., 2020) and/or avoidance of worry by circumventing potential triggers, which may include medical appointments (Butow et al., 2018). Without treatment, for many people, FCR remains present over time, even after 10 years (Gotze et al., 2019).

Almost all cancer survivors who experience FCR need support and for 40% this includes psychological care (Luigjes-Huizer et al., 2022c). Several effective interventions have been developed, mostly consisting of specialized face-to-face treatments provided by psychologists (Cincidda et al., 2022). However, due to the large amount of cancer survivors, resources and staff are not available to offer specialized mental healthcare to all. Moreover, many patients with FCR may not need or want intensive psychological treatment (Butow, 2021). Stepped care or matched care models, in which care is provided based on FCR severity, have been recommended, reserving resource-intensive specialist care for those with the most severe FCR and providing low intensity types of support (e.g. nurse-led interventions) to those with mild to moderate FCR (Liu et al., 2019; Pradhan et al., 2021).

Because general practices have an increasing role in survivorship care and are used to providing easily accessible care for moderate psychosocial complaints, they may be the right actor to provide these low intensity types of support for FCR. Cancer survivors frequently favor their general practitioner (GP) for psychosocial care and GPs also consider this a role that fits their position (Deckx et al., 2021). However, GPs often do not systematically screen for psychosocial issues and few interventions are available for GPs to manage the psychosocial problems of cancer survivors (Deckx et al., 2021).

In our study, a primary care intervention for cancer survivors with FCR was developed. It consists of an e-Health program and three to five 30-minute sessions with a primary care based mental health worker (MHW, POH-GGZ in Dutch). It is one of the first eHealth interventions for FCR that is not psychologist-led, and also one of few interventions that is designed to be low-resource and easily accessible. To evaluate this intervention, two RCTs have been conducted known as the BLANKET study. In the first RCT, sessions with the MHW were held face-to-face at patients’ own primary care practice (Luigjes-Huizer et al., 2019). However, the number of participating patients per practice was low and due to the Covid-19 pandemic, no new practices could be included. Therefore, inclusion was stopped before the required sample size was reached (Luigjes-Huizer et al., 2022a). Considering that patients do not need GP involvement to opt for the intervention, a second RCT based on self-referral recruitment was started. The sessions were provided via video calling by MHWs specifically employed for the study, who were not part of participants’ regular primary care team. The intervention was found to be effective in decreasing FCR and in improving general mental well-being, and this effect remained at 10 months follow-up (Luigjes-Huizer et al., 2023).

As a final step toward clinical implementation, the intervention’s feasibility and acceptability were qualitatively assessed, based on patients’ and MHWs’ perspectives. The aim was to investigate how patients and MHWs experienced the intervention. Furthermore, since meta-analyses have shown that guidance usually increases interventions’ effectiveness (Baumeister et al., 2014; Domhardt et al., 2019), a second aim was to investigate the essentiality of the role of the MHW. Finally, the third aim was to investigate perceptions on primary care as the setting for this intervention.

## Materials and methods

### Design

In this qualitative study, we conducted semi-structured interviews with nine patients and thirteen MHWs who had participated in one of the two RCTs of the BLANKET study (Luigjes-Huizer et al., 2022a, 2023).

### Study population and recruitment

The study population of both RCTs consisted of adult cancer survivors who (a) finished successful curative cancer treatment between 3 months and 10 years ago, (b) wanted support for FCR, and (c) had sufficient Dutch reading and writing skills. In the first trial, survivors were recruited by letter via participating GP practices. In the second trial, they were recruited online via social media, cancer patient organizations, and existing cancer cohorts. In the patient information letter they received, they were informed they might be approached for the interview study.

After trial participation, patients and MHWs were contacted via e-mail and/or phone to recruit them for the interview study. Written informed consent for recording and analyzing the interviews was provided prior to the interviews. MHWs from both trials were interviewed. Unfortunately, due to time and resource constraints only patients from the first trial were interviewed, and not from the second trial. In that regard, we were unable to purposively sample our participants.

### The intervention

The primary care intervention was designed at the Helen Dowling Institute, an academic mental health institute specializing in psycho-oncology. It includes an e-Health program with three CBT-modules and five optional modules on rumination, avoidance, relaxing, reassurance, and undertaking activities, which can be selected based on patients’ individual needs. The program includes information, exercises, and videos with experiences of other patients, and is available 24/7. Patients also have three to five 30-minute sessions with a trained MHW.

### Data collection

The semi-structured interviews were conducted by YL and AR, who have been professionally trained in qualitative research. Interview guides were used. They were designed by the research team and improved after three interviews. The guides contained questions about expectations, experiences, positive and negative aspects, practical concerns, the primary care setting and an overall appraisal (see Supplemental Materials). Neither interviewer knew the interviewees before study participation. Interviewees were aware that the research was being conducted to improve the care for patients with FCR and was part of a PhD dissertation. No one else was present during the interviews. Interviews were audio-recorded and transcribed verbatim. In addition, brief notes were taken by the interviewers. As the content of the interviews was rather straightforward, no member checks were carried out.

### Qualitative data analysis

The transcripts were analyzed using inductive thematic analysis. We used the phases outlined by Braun and Clarke to structure data analysis (Braun and Clarke, 2022). In the first and second within-case analysis phases, two researchers (YL with RW or AR) independently familiarized themselves with the transcripts and conducted initial open coding of the interviews using MAXQDA software (“VERBI Software, MaxQDA”, 1989–2020). Subsequently, they discussed the codes until reaching consensus. This led to a coding scheme on which the analysis of the following interviews was based. After the first five interviews of each group (patients, MHWs from trial 1, and MHWs from trial 2) were analyzed, YL coded the remaining interviews. After all interviews were analyzed, two researchers (YL with RW or AR) organized the codes into potential topics according to the third cross-case analysis phase, which were checked with the interview data in the fourth phase. During the fifth phase, the multidisciplinary research team (ML, CH, SD, MS, and YL) held two meetings and grouped the codes and topics into themes. In the sixth and final phase, the manuscript was written.

### Research team

Six of the eight authors are women. YL is a health scientist and epidemiologist. MS is a behavioral scientist and senior researcher in psycho-oncology. SD is an epidemiologist and PhD candidate. RW is a behavioral scientist and junior researcher in psycho-oncology. AR is a research assistant. NW is professor of general practice with 25 years’ experience as GP. CH is MD, epidemiologist and assistant professor of general practice. ML is professor in medical psychology, and a healthcare psychologist with extensive experience in treating FCR.

### Ethical statement

The Medical Research Ethics Committee (METC) Utrecht issued approval of the study with number 18/879, together with their approval of the RCTs.

## Results

### Participants

Nine out of 13 participants who received the full intervention in the first trial, were approached and interviewed. The remaining participants were not selected, because they were treated by the same MHWs as other interviewees. Participants from the second trial could not be interviewed due to time and resource constraints. Six out of eight MHWs from the first trial and seven out of eight MHWs from the second trial were interviewed. The remaining MHWs declined to participate, because of limited experience with the intervention, a preference not to be audio recorded, and a lack of time. All interviews took place between December 2019 and November 2021. Patients were interviewed at the location of their preference (patient’s residence (*n* = 6), the Helen Dowling Institute (*n* = 2) or by phone (*n* = 1)) and lasted approximately 1 hour. The interviews with the MHWs lasted 45 minutes and were conducted at the GP practice (*n* = 2), via phone (*n* = 4), and via video calling (*n* = 7). In Table 1, sociodemographic and clinical characteristics of the patients are presented, which are comparable to the whole sample. In Table 2, sociodemographic and professional characteristics of the MHWs are presented.

**Table 1.** Sociodemographic and clinical characteristics of the nine patients.

Characteristics	Patients (*n* = 9)
	Mean (range)
Age (years)	59.2 (39–76)
Time since diagnosis (years)	4.9 (1–9)
Time since end of treatment (years)	4.8 (1–9)
FCR at baseline (FCRI-SF*, range 0–36)	22.3 (19–26)
	*n* (%)
*Gender*	
Men	4 (44)
Women	5 (56)
*Cancer type*	
Breast cancer	4 (44)
Respiratory system	2 (22)
Hematological	1 (11)
Colorectal	1 (11)
Liposarcoma	1 (11)

* FCRI-SF: Fear of Cancer Recurrence Inventory, short form.

**Table 2.** Sociodemographic and professional characteristics of the 13 MHWs of trial 1 and 2.

	MHW 1–6, trial 1 (*n* = 6)	MHW 7–13, trial 2 (*n* = 7)
	Mean (range)	Mean (range)
Age (years)	43.3 (26–56)	52 (24–65)
Working experience (years)	n.a.	18.9 (0–30)
Number of patients seen	2 (1–3)	20.4 (4–46)
	*n* (%)	*n* (%)
*Gender*		
Men	0 (0)	1 (14)
Women	6 (100)	6 (86)
*Educational background*		
Psychology	4 (67)	2 (29)
(Social psychiatric) nursing	2 (33)	3 (43)
Social work		1 (14)
Other		1 (14)

### Feasibility and acceptability of a primary care FCR intervention

The analysis of the interview data resulted in five main themes: (1) *treatment content*; (2) *treatment support*; (3) *treatment format*; (4) *patient characteristics*; and (5) *primary care setting*. Quotes belonging to each theme have been included.

Overall, both patients and MHWs appreciated the program. While some patients still experienced fear after participation, they were better able to manage it. Patients described that the intervention helped them find new coping strategies, process their emotions, and learn to involve their support system when needed.

### Theme 1. Treatment content

#### Focusing on cancer

Both patients and MHWs valued the content of the intervention. MHWs explained that in daily practice, they already had modules to treat fear. However, patients and MHWs appreciated that this program was specifically focused on cancer related fear, including videoclips of other patients sharing their experiences. Patients recognized themselves in these experiences and felt supported.

Everything she [the patient] read in there, she recognized, and she felt the same things. She received a bit of reassurance, a bit of confirmation that what she was doing, was okay. And also like, it’s not strange that when you see a commercial, that it can trigger fear. The goal is not to completely let that disappear, that you’re not allowed to be fearful anymore. MHW 1, female, age 26.

#### Stimulating self-management

Doing the program confronted patients with their FCR, which they experienced as difficult yet helpful. They described how the program helped them to reflect on their thoughts and feelings, and to apply the learned coping strategies when needed.

I remember sitting here and opening the program and that it really moved me. That it made me really sad. And those were the moments you start thinking about it again. A sort of learning moment. And not even that I really benefitted from what was being said, simply that it forced me to look at myself. Patient 1, male, 43.

#### Fitting the intervention to patients’ needs

MHWs indicated that the intervention is useful for different types of people because it can be flexibly adapted to each individual patient. Different optional modules suited different patients. Also, MHWs described that the number and depth of the conversations differed between patients, matching their needs.

There’s not just one thing that helps, all those different modules can be helpful. I always say, read through the program and get out of it what is of benefit to you. And that really differs. MHW 12, female, age 61.

### Theme 2. Treatment support

#### Open communication

MHWs created a safe environment for patients to share their stories and express their thoughts and feelings. Patients appreciated being heard. MHWs and patients emphasized the importance of establishing a good relation for the sessions to be supportive.

Those were some very good conversations. Very nice for me to talk about things that I normally don’t express. Patient 6, male, age 71.

#### Facilitating role

MHWs and patients described how the MHWs clarified and elaborated on the topics in the program. For example, they probed patients how to best apply what they learned in daily life. Also, MHWs encouraged patients to do the exercises and to keep going.

I ask, ‘Oh yes, and how was that?’ Then I try to adapt to what comes next. And then I always ask, ‘Did you also work with the modules? And how did that go? And how could that help you in this situation?’ MHW 4, female, age 46.

#### Prior experience with cancer

Both patients and MHWs indicated it was very helpful that the MHWs had prior knowledge on cancer, its treatment, and its psychological consequences. This helped them to acknowledge and normalize patients’ experiences, and prevented them from being scared or shocked by patients’ stories. Some patients shared that, prior to this study, they had encountered an MHW in daily practice that was unequipped to treat FCR.

I just like it better when I have a good sounding board. In the sense that people really know what they are talking about. And of course, the general practitioner also has that, but the general practitioner cannot talk to you for half a year, for half an hour. Patient 9, female, age 54.

#### Including the support system

While it was not a standard aspect of the intervention, patients and MHWs also highlighted the benefit of MHWs considering the patient’s support system. When the patient has a partner, he or she can comfort the patient after emotional sessions. Partners can also help the patient manage FCR in daily life. If there was no partner, MHWs discussed whether patients were comfortable doing the online program without support at home and helped patients to identify people to contact if needed.

What I noticed so far is that partners are often involved. That is also what I advise, involve your partner or a good friend. So that when things are difficult, you have someone that you can call. I always try to check whether the support system is sufficient to do that. MHW 3, female, age 47.

### Theme 3. Treatment format

#### Treatment format: Online program and sessions with MHW

Patients and MHWs considered the *combination* of an online program and (face-to-face) sessions with an MHW of added value. The online program was continuously accessible and allowed patients to work at their preferred pace and time. The sessions with the MHW provided space for patients to share their stories and feel supported (see Theme 2. Treatment support). Patients indicated that without sessions, the program would feel too impersonal, and it would be difficult to engage with this sensitive topic, whilst only having sessions would take too much time for both MHWs and patients.

But if you only have those exercises, then you are just doing that on your own. […] And you already feel incredibly alone, and then you also need to do those exercises. While you’re really scared. Patient 1, male, age 43.

When we look at those long waiting lists, I think that working online can definitely be a plus for people to start working on it by themselves. That way, help can be offered to them sooner, but it is also nice for us because it takes some pressure off your schedule. MHW 3, female, age 47.

#### Online sessions with MHW

Online and face-to-face sessions each have pros and cons. MHWs indicated that online sessions take less time, fit more easily between other activities, can be offered to patients who live far away, do not involve a risk of COVID-19 contamination, and can even continue when patients feel sick. MHWs learn about patients’ home situation and patients feel more human and less like patients. MHWs expressed that they were able to build a relation with patients using video calling, as they do in face-to-face sessions. A disadvantage of online sessions is that video calling sometimes falters due to bad internet connections and that for some, especially older, patients it was unfamiliar and somewhat stressful. Also, when distressing events occurred (e.g. patients developing metastases), online contact could feel quite distant and some MHWs considered it insufficient to support patients the way they wanted. Importantly, some patients also strongly prefer face-to-face sessions.

Despite only having sessions via video calling, you do build a connection with each other. And yes, it doesn’t really matter whether you see each other physically or not. MHW 7, female, age 65.

#### Face-to-face sessions with MHW

The main benefit of face-to-face sessions is the increased depth of conversation. Also, there are no distractions from the home situation (e.g. children), and MHWs noticed that being required to travel to sessions ensured patients’ motivation for treatment. Some MHWs considered face-to-face sessions more appropriate for the sensitive subject, and some found it easier to motivate patients who avoid as a coping strategy during face-to-face sessions. Some MHWs also indicated that lonely patients feel more supported by face-to-face contact. MHWs suggest a combination of online and face-to-face sessions may be ideal, for example, having the first and last sessions face-to-face and the others online.

A conversation with someone you can look directly in the eyes, is of course the very best. Patient 8, female, age 54.

### Theme 4. Patient characteristics

While MHWs considered the intervention useful for many different patients, there were also patients for whom it seemed less suitable.

#### Lacking digital and language skills

To benefit fully from the program, digital and language skills are required. A few patients had trouble with the online modules. Also, some patients had trouble expressing themselves and writing down what they felt.

Language can be quite hard. That’s why those videoclips are so good, because it [rest of the program] is quite focused on language. That is a disadvantage, I think. So then, such a thought record [a type of exercise] can be quite difficult. So if I didn’t want that for people, then I only worked with the basic modules. MHW 11, female, age 51.

#### Prior knowledge

While for some the content was too challenging, for others it was too simplistic or familiar, decreasing their willingness to do the program. The MHWs adjusted how quickly they went through the modules, but this was not enough to engage some participants.

You cannot offer a course on different levels; this is of course for everybody. […] You often have to maintain a basic skill level. But when you don’t have that level [when your level is higher], it starts to become annoying after a while. It becomes too explanatory, too stimulating, too emphasized, especially too emphasized. Patient 2, female, age 76.

#### Lack of time or motivation

MHWs reported that despite their support, a few patients lacked time or motivation to engage with the online modules in between sessions. Some patients described having only moderate fears and felt the program and the time it requires was therefore out of proportion. Yet, others felt that, despite having moderate fears, it made a positive and important difference in their lives.

She quit. She was not motivated. She had a neighbor who said, ‘you should do this, too, it’s very good for you’. It turns out that’s not the right motivation. MHW 7, female, age 65.

### Theme 5. Primary care setting

#### GPs provide holistic and accessible care

Our intervention was situated in the primary care setting. MHWs and patients indicated this was fitting because GPs can easily check in with patients, especially when hospital care has ended. GP care is accessible and nearby patients’ homes, and many patients have longstanding relationships with their GPs, which provide a safe space to discuss FCR. MHWs explained that when patients often present with physical symptoms and no physical issues are found, GPs can discuss a potential relation with FCR and, if relevant, offer psychological care. They also described that compared to hospital or specialized psychological care, GP care helps patients to feel more normal and less like patients, which patients prefer, and which is helpful for their recovery.

A three-month oncological revalidation program is much more intense, with much more personal attention and context, but if that barrier is too high for people, this can really help. Not everyone wants to travel to such a program or is mobile enough. Or people can find it too much and too intrusive, then this might help much more. Patient 3, female, age 39.

#### Barriers in discussing FCR with GPs

GPs have limited time and see many patients. Relatively few of them suffer from FCR. Some MHWs explained that considering the large number of topics GPs provide care for, they may not prioritize FCR. Some patients described they barely know their GP or do not have a positive relationship and would therefore not feel comfortable sharing this sensitive topic. In general, many patients would not mention FCR to their GP and appreciate it if the GP proactively discusses it. However, since this often does not happen, MHWs stressed the importance of informing patients about FCR, so that they can seek help when they cannot manage FCR on their own.

I really hope this gets implemented at the GPs. I really think it is very valuable. And at the same time, I hope GPs get a bit more financial space because that’s the other side. GPs get more and more responsibilities, right? And always [require] more resources so to say. It is not our problem, but of course they should also get some financing. Like, who will do what? But I think this is a very valuable type of care, that does not have to cost that much. MHW 12, female, age 61.

#### Fit with MHW role in GP practices

MHWs indicated that within the GP practice, the intervention fits well with the role and expertise of MHWs, who offer short-term, accessible interventions, including CBT and e-Health programs. In our study, all MHWs were trained. They found it helpful to practice listening and being present without immediately trying to solve patients’ problems. This helped MHWs to manage their feelings of powerlessness. The information about (the influence of) cancer was also new and helpful. Some MHWs stated MHWs do not require specific training to provide this intervention, especially if the topic is already touched upon in their education. Other MHWs posited that the topic is very intense and that some MHWs lack CBT skills. They therefore recommend specialized training. Moreover, if MHWs regularly provide the intervention, it helps them to offer It smoothly.

You work with people who had a life-threatening illness, you need to offer CBT, and you need to know some things about psychopathology. So I think these three things really need to come together. I don’t think this can be done by just anyone. For that, the target group has been through too much already. MHW 9, female, age 40.

## Discussion

In this study, we have shown that the primary care CBT-based intervention for FCR, which was previously found to be effective in reducing FCR (Luigjes-Huizer et al., 2023), is also positively valued by patients and MHWs. The intervention helped patients learn how to manage their fear. The online program was appreciated for being recognizable, adaptable and time efficient, and stimulating self-management. The sessions with the MHWs engaged patients, motivated them to continue, provided a safe space to share stories, and helped them to include or broaden their support system. Since a prior RCT found that the e-Health program was ineffective without support (van Helmondt et al., 2020), in line with other studies on the ineffectiveness of e-Health programs without support (Baumeister et al., 2014; Domhardt et al., 2019; Wu et al., 2025), the role of the MHW seems to be essential for the intervention’s effectiveness, feasibility and acceptability. Without support, patients may not engage with eHealth interventions (van Helmondt et al., 2020) and also miss the opportunity to be heard (Cincidda et al., 2022).

For some groups, the program appeared less suitable, namely patients lacking digital or language skills and those who considered the content too simplistic. Both patients and MHWs considered the intervention very suitable for the primary care setting, since it fits in GPs’ holistic perspective that considers both physical and psychosocial complaints, and matches with MHWs’ role of offering accessible short-term interventions. Online and face-to-face sessions each have advantages and disadvantages. Ideally, the patient can combine both as preferred.

### Strengths and limitations

A strength of our study is that we interviewed both patients and MHWs. A limitation is that we were unable to include patients from the second trial. Fortunately, we were able to include MHWs who gave information about the patient perspective, but data saturation likely was not reached on the patient perspective on receiving the intervention via video calling. We do not expect this to affect the overall conclusion on the intervention’s feasibility and acceptability, especially for implementing it with live sessions, since interview data from patients from the first trial and questionnaire data from patients from the second trial show that patients find the intervention feasible and acceptable (Luigjes-Huizer et al., 2023). Also, previous meta-analyses have shown that digital interventions, including CBT and blended interventions, are effective in reducing FCR (Cindidda et al., 2022; Kang and Yu 2023; Zhang et al., 2025). We may have missed some potentially valuable data on the patient experience, which could have further enhanced the intervention and its implementation via video calling. However, since prior research on care via video calling during the COVID-19 pandemic has shown that, while patients experience somewhat more distance and somewhat less personal contact online, they are pleasantly surprised about the quality of the therapeutic relationship and feel more at ease at home (van der Lee and Schellekens, 2020), we anticipate that most patients will not perceive receiving the intervention through video calls as an impediment to its acceptability or feasibility. Also, when implementing the intervention in primary care, MHWs can offer face-to-face sessions to their patients and can discuss with patients which mode is most suitable for them.

### Implications for clinical practice and research

Because of the intervention’s previously demonstrated effectiveness, its acceptability, and the good fit with primary care, we recommend implementing it in primary care, where it can provide an affordable, accessible alternative that complements existing specialized care, as has been called for (Liu et al., 2019). For some patients, especially those with mild to moderate FCR, this intervention may be sufficient, and it may prevent developing more severe FCR. For others, it may serve as a helpful starting point, especially if there are waitlists for specialized care. The blended approach that combines the benefits of both online and face-to-face components seems particularly suitable for those with mild to moderate FCR, because it offers sufficient intensity of the intervention and therapist guidance to increase adherence and provide space to be heard, while the flexibility of this approach, along with reduced travel time and costs, aligns with the investment patients are willing to make based on their level of FCR.

However, there are practical barriers for implementation in primary care. There are few patients with FCR per GP practice, making it difficult for MHWs to build expertise and possibly limiting the investments practices are willing to make to provide this care. Therefore, MHWs need to learn about FCR as part of their regular professional training and the online program needs to be easily accessible (e.g. included in widely used e-Health platforms), so that MHWs can easily implement it when they encounter a patient with FCR. Additional training can be offered for MHWs who feel uncomfortable with the topic or who want to specialize in this topic. If, in a different context, there are no MHWs available in primary care, the intervention could likely also be guided by a nurse. In previous studies, nurses have effectively provided FCR interventions (Liu et al., 2019). Still another option for implementation could be for nurses or MHWs working in hospitals to provide this intervention. We do not recommend implementing it without any support, because it was found to be ineffective as a self-help intervention (van Helmondt et al., 2020).

It is also important that the available care reaches patients. A recent feasibility study identified lack of FCR awareness among patients and inadequate detection and referral by healthcare providers as barriers for FCR interventions (Deuning-Smit et al., 2022). Since patients often do not take initiative to discuss FCR with their doctor, it is helpful if healthcare providers proactively tell patients that they may experience FCR, and that care is available. Therefore, increased awareness among GPs is needed. Also, future research could investigate the best phase and ways to inform patients about FCR and the available care options.

For some patients, this intervention seemed less suitable. This includes, for example, patients who lacked adequate digital skills, had trouble writing about their thoughts and feelings or who considered the content too simplistic or familiar. By adjusting the program to patients’ needs and preferences, MHWs can increase its suitability and utility. They can, for example, provide the program on paper, do the first exercises together, or encourage patients to *apply* already familiar strategies in daily life. In doing so, the program is expected to be helpful to a large proportion of cancer survivors experiencing FCR. Still, it is recommended to discuss beforehand with patients whether the intervention matches their needs. Also, making interventions less language-dependent, for example, by adding more videos, could improve their usability (Deuning-Smit et al., 2022). Finally, research is needed on factors that predict which patients require professional help and what kind of help works best for whom.

### Conclusions

Patients and MHWs appreciate the primary care FCR intervention, which combines an online program with (online) sessions with an MHW and was previously shown to reduce FCR severity. We recommend implementing it in primary care, by making it available on all existing e-Health platforms for MHWs, including FCR in general education for MHWs and ensuring all patients and healthcare providers receive information about FCR and the available care types.

## Supplemental Material

sj-docx-1-hpq-10.1177_13591053251345579 – Supplemental material for A qualitative study on the feasibility and acceptability of a primary care intervention for fear of cancer recurrence Supplemental material, sj-docx-1-hpq-10.1177_13591053251345579 for A qualitative study on the feasibility and acceptability of a primary care intervention for fear of cancer recurrence by Yvonne L Luigjes-Huizer, Melanie PJ Schellekens, Niek J de Wit, Charles W Helsper, Sophie I van Dongen, Anne S van Roozendaal, Rosalie AM van Woezik and Marije L van der Lee in Journal of Health Psychology
