# Supplementary material for: A qualitative study on the feasibility and acceptability of a primary care intervention for fear of cancer recurrence
Source: J Health Psychol. 2025 Jul 10;31(3):1152–63. doi: 10.1177/13591053251345579 (PMC12949749; doi:10.1177/13591053251345579)
Supplement: sj-docx-1-hpq-10.1177_13591053251345579 – Supplemental material for A qualitative study on the feasibility and acceptability of a primary care intervention for fear of cancer recurrence [file sj-docx-1-hpq-10.1177_13591053251345579.docx]

# Interview guides on the feasibility and acceptability of a primary care intervention for fear of cancer recurrence

## Interview guide for patients

*Complaints*

1. Before we talk about the support you received: Can you tell us something about the complaints you had when you decided to participate in the study?

- Anxiety/worries about cancer recurrence, ruminating
- Sleep problems, physical complaints, tension
- Work, social contacts, going out, family, household

*Needs*

1. Can you tell us something about the expectations you had of the guidance you would receive?

- Positive expectations
- Doubt

*Experiences*

1. Can you briefly describe the intervention you received?

- Mental health worker/general practitioner/referral
- Online program

1. What was it like for you to receive the intervention?

- Positive experiences, explanation
- Negative experiences, explanation
- Make sure that it remains clear whether it concerns the general practitioner / MHW / online programme

1. What do you think helped and why?

- Which aspects? and how/why? what helped the most?

1. What didn't help and why?

- Intervention components that had no effect
- Missing parts
- Not the right intensity/setting
- Right timing

1. Can you tell us something about how you experienced the contact with the GP (in the context of this intervention)?

- How was the contact?
- How is the contact in general?

1. Can you tell us something about how you experienced your contact with the MHW (in the context of this intervention)?

- How was the contact?
- Had you had contact before?

1. If applicable, what was it like to do the online program?

- Which parts had added value and why? (show summary of components)
- Which parts had no added value and why?
- Did everything work, or did something sometimes not work?
- What did you think about the online platform?
- What made it convenient or inconvenient to use?

1. What did you think of the exercises?

- Did the exercises help you? With what and how? (ask for examples)
- What have you learned?
- Are you still doing the exercises? When? Which?

1. Was the care you received useful to you? Why or why not?

- Travel time and costs
- Time and location of care
- Availability/speed of feedback
- Clarity of agreements made (appointments etc)

1. Do you feel that the intervention has helped? How do you notice that?

- Fear
- Sleep problems, physical complaints, tension
- Work, social contacts, going out, family, household

1. Did you receive any other forms of support during the same period that made a difference to you? Which kinds? How was it helpful?

- Care from family/friends
- Books
- Religious/spiritual support

*Ending the interview*

1. Can you briefly indicate to what extent this intervention is appealing and what the added value is according to you?
2. Is there anything else you would like to share?
3. What was it like for you to participate in the study?

## Appendix A

## Overview of modules of the online program

Two basic modules

1. Psycho-education: recognizing fear
2. Basic principles of CBT (part 1 and 2)

Five optional modules

1. Rumination
2. Avoidance
3. Undertaking enjoyable activities
4. Learning to relax
5. Reassurance

## Overview of exercises

Recognizing signals of fear

Recognizing situations that cause fear

Charting feelings, thoughts and responses to situations that cause fear

Recognizing unhelpful thoughts, challenging these thoughts and thinking of helpful thoughts

Thoughts-stop technique

Rumination fifteen minutes

Rumination elastic

Not thinking but doing (something else)

Charting situations that you avoid and breaking through avoidance

Undertaking enjoyable activities

Learning to relax

Reassurance strategies

## Interview guide for MHWs

*Questions about the training*

## What was it like to participate in the training?

- What was of added value? What was not? Why?
- What points for improvement do you see? Why?
- New things learned
- Duration
- Right aspects: knowledge, self-efficacy, practicing with actor
- Is it a topic that has priority for you?

*Questions about the guidance*

## Has your way of treating patients changed since you participated in the training?

- Example / explanation

## Can you briefly describe the treatment you provided to your patients?

- Number of sessions, topics
- Online program

## What was it like for you to provide the treatment?

- Positive experiences
- Negative experiences

## When you think about your patients who have received treatment, do you feel that the treatment has had an effect? Do you notice a difference compared to before the treatment?

- Fear
- Sleep, physical complaints, tension
- Work, social contacts, family, household

## In what way and through which aspects did the treatment have added value in your opinion?

- Which aspects? and how/why?
- Psycho-education, normalization, self-management
- Show a list of parts and exercises and ask what worked and what did not

## In what way did the treatment not help?

- Intervention components that had no effect
- Missing parts
- Not the right intensity/setting

## Was the care you provided practical for you and your patient?

- Time investment
- Appropriate intensity of contact
- Clarity of agreements
- For people who had five sessions: could it have been less for those patients?
  For those who had less sessions: was five too much?

## What is your opinion of the online program offered?

- Complete? Better than alternatives?
- What did you think of the online platform?
- Was it convenient to use?

## Do you think that this type of care and this issue is appropriate for the general practitioner and the MHW (either online or face-to-face)?

- Does it fit with the relationship patients usually have with their GP? (in your experience)
- Is the subject too vulnerable?
- Did you feel sufficiently equipped? Would you have felt equipped without the training?

## *For part B only*: How did you feel about offering this treatment online, to patients you do not know?

- Is it appropriate for the subject?

*Ending the interview*

## In summary, do you think this form of care by the MHW (either online or face-to-face) is appealing and has added value?

- Follow-up question: Are there groups for whom it is not appealing or of added value? Why?
- For which people do you think this works especially well? Why?
- Which people would you not offer this? Why?
- Do you see any points for improvement for this type of care?

## What possibilities do you see for offering this type of care in daily practice?

## Is there anything else you would like to share?

## What was it like for you to participate in the study? Why did you choose to participate?
